# A District Nurse and Her Patients

**Published:** 1886-12-18

**Authors:** 


### A District Nurse and her Patients.

By the Matron of an East London District Nursing Society.

I am a Matron of a large Nursing Society, and my work

**Our Work.** lies in the districts of Stepney, Whitechapel, and Spitalfields; very different work it is from hospital work, where one has everything one needs for the comfort and recovery of the patients. But, though sorely incommoded for want of suitable appliances, and sometimes even for the necessaries of life, district nursing is not without its redeeming features. In no other field does one get a better insight into the domestic relations of the poor, and there is an alloy of comfort to reward our exertions in the gratitude our patients display, and in the confidence and hopeful feelings inspired by our efforts on their behalf. My duties bring me into intimate communication with all sorts and conditions of men, women, and children, and the stories they have to tell of their hard struggle with life are often interspersed with amusing episodes, which impart a silver lining to the dark clouds which too often envelope their history. One of the most important of our Society rules requires each nurse to report new cases at once to the Matron, and that she shall visit them as soon as possible, to give instructions, and to see that the doctor's orders are faithfully carried out by the nurses.

One day, Mahoney of Skittle Alley is announced to

**My Patient.** be suffering from rheumatism and extreme debility. Luckily, I can visit him the same day, and on reaching his lodgings I recognise a well-grown lad of about eighteen, of frank expression, dark curly hair, and blue eyes; such eyes! as only the Irish can boast of. He is evidently embarrassed, and can hardly tell the too oft-repeated tale: Oh, yes, nurse had been very kind; he had a good doctor. The pains lay chiefly in his head. His work had exposed him to all weathers, and he had broken down under it, and did not know how the landlady could be paid. The latter, who accompanied me to the door, volunteers some further information: the lad had been out of work more than five weeks, and had literally starved himself to pay his rent. At last he got work, and was as pleased as any child when he heard of it, and craved for strength to enable him to do it; but, alas! his health gave way, and so he became our patient. We sent him nourishment for a few weeks, and a lady, to whom I mentioned the case, most kindly provided him with flannel shirts and warm stockings. He has now resumed his work, and is likely to be employed all the winter, should his health continue as it is at present.

I proceed on my journey to the next parish, to visit a

**A Sick Child.** sick child; we call her little Jeannie. She is suffering from dropsy and heart-disease, and has been ill for many months, the weary, sad face revealing an age of suffering. She is only seven, and whilst she lies on her rude though clean little bed under the window, she hears children of her own age laughing and shouting in high glee, without a care; but this tiny thing is too weary even to smile. A lady who sometimes accompanies me in my visits, full of tender sympathy when she saw the child, had kindly sent me a doll for her, which I carefully hid under my cloak to give her a glad surprise. When I entered, the mother told me, with tearful eyes, that her poor little girl had spent a dreadful night. I sat down as usual by the bed, and tried to draw her into conversation. "Jeannie, do you remember that nice lady I brought to see you the other day?" "Yes, Matron." "Well, do you know that a little bird whispered to her that Jeannie was going to have a birthday soon, so she has sent her,"—bringing from its hiding-place, "a dollie!" The two little wasted hands were stretched out, oh! so eagerly, to grasp the welcome gift, and the little worn face lighted up with a beam of pleasure which I had never seen on it before, impossible to describe, but everything to witness. We christened it on the spot; and when her good mother carries her to the out-patient room of the hospital, she turns her head languidly and says: "Mother, is Clara ready?" and so forgets the doctors.

My next visit is to an old woman of 79. I have seen

**Grannie.** her, of course, before, for she has just recovered from an attack of bronchitis, but is as well now as she ever will be in this world. She tells me she can "muddle" along again by herself as she used to do before her illness, now that she feels stronger, and is much obliged to us for all our kindness. She appears quite happy and contented, and is certainly independent of our help and that of her neighbours; but she dearly loves a gossip, and is very pleased with any little offering. Although the day was far advanced, I found, on calling, that Grannie, as we call her, was still in bed, so I grope my way in the dark, up a narrow and rickety staircase, to a small back-room, sadly devoid of furniture, and worthy of a descriptive illustration by G. R. Sims. As I muse on the miserable aspect of the room, and wonder what I should have done if my own dear mother had ever been reduced to such a plight, a cheerful voice welcomes me, with a "God bless you, my dear! so you've come in again to see me. I ain't up, do you see, but I'll be a-gettin' up now, direckly." "Oh, Grannie," I say, "lie still until I try to light a fire, for it is so cold to-day, and you will get a chill." "Nonsense, my dear; I'm used to it, and I'm sure you can't kindle a fire in that 'ere grate."

Now, if I pride myself on anything it is that of being

**Vanquished.** able to make a fire burn on the shortest notice; so, suiting the action to the word, I commence clearing the ashes out of the grate. I look round for wood, and wood there is, but only in the shape of a chump from the root of a big tree. I espy a chopper in the corner of the room, and with this I make an earnest attempt at chopping off sticks; but it is all in vain: the wood is tough and green. Almost in despair, I think I will ask some other woman in the house to give or lend me some sticks, when, a step behind me, I see Grannie, attired in an old petticoat and a rusty black shawl, pitying my efforts. "Move a one side, Mum," she says. "I can't lay abed and see you a-striving with that chopper; you ain't used to it, and ain't strong in the wrists, neither." She took the chopper from my hands, and soon contrived to rend asunder the tough piece of wood, and in a few minutes we had a bright little fire blazing. I felt utterly crestfallen, for Grannie's superiority in fire-lighting was a new experience to me; and, before wishing her good-bye, I offered to make her bed, out of very shame, and you may be sure I devoted my best efforts to the task.

Christmas is at hand, and how I am to get through

**In Brief.** the cold weather and relieve my poor people is a mystery; perhaps some generous reader may help me to solve it. The Editor of The Hospital will receive and forward any contributions entrusted to his care.

